# A Septin from the Filamentous Fungus *A. nidulans* Induces Atypical Pseudohyphae in the Budding Yeast *S. cerevisiae*

**DOI:** 10.1371/journal.pone.0009858

**Published:** 2010-03-25

**Authors:** Rebecca Lindsey, Youngsil Ha, Michelle Momany

**Affiliations:** Department of Plant Biology, University of Georgia, Athens, Georgia, United States of America; CNRS UMR6543, Université de Nice, Sophia Antipolis, France

## Abstract

**Background:**

Septins, novel cytoskeletal proteins, form rings at the bases of emerging round buds in yeasts and at the bases of emerging elongated hyphal initials in filamentous fungi.

**Methodology/Principal Findings:**

When introduced into the yeast *Saccharomyces cerevisiae*, the septin AspC from the filamentous fungus *Aspergillus nidulans* induced highly elongated atypical pseudohyphae and spore-producing structures similar to those of hyphal fungi. AspC induced atypical pseudohyphae when *S. cerevisiae* pseudohyphal or haploid invasive genes were deleted, but not when the *CDC10* septin gene was deleted. AspC also induced atypical pseudohyphae when *S. cerevisiae* genes encoding Cdc12-interacting proteins Bem4, Cla4, Gic1 and Gic2 were deleted, but not when *BNI1*, a Cdc12-interacting formin gene, was deleted. AspC localized to bud and pseudohypha necks, while its *S. cerevisiae* ortholog, Cdc12, localized only to bud necks.

**Conclusions/Significance:**

Our results suggest that AspC competes with Cdc12 for incorporation into the yeast septin scaffold and once there alters cell shape by altering interactions with the formin Bni1. That introduction of the *A. nidulans* septin AspC into *S. cerevisiae* induces a shift from formation of buds to formation of atypical pseudohyphae suggests that septins play an important role in the morphological plasticity of fungi.

## Introduction

Septins were first discovered in *S. cerevisiae* where they form a scaffold that organizes the bud site and are a component of the morphogenesis checkpoint that coordinates budding with mitosis [1], [2], [3]. Five of the seven *S. cerevisiae* septins (Cdc3, Cdc10, Cdc11, Cdc12 and Shs1) co-localize as a ring or collar to the neck region where the daughter bud emerges from the mother cell. At least 35 other proteins, including cell wall biosynthetic enzymes and cell cycle regulators, localize to the bud neck region in a septin-dependent manner [4], [5]. Mutation of any of the core septins, *CDC3, CDC10, CDC11*, or *CDC12*, prevents formation of the septin ring, resulting in elongated buds and mitotic delay [2]. During sexual reproduction, the Spr3 and Spr28 septins localize with Cdc3, Cdc11 and Cdc12 at the leading edge of the prospore membrane as it encapsulates the nucleus [6], [7], [8]. Cdc12 occupies the central position of the core heteropolymeric septin complex in *S. cerevisiae*, binding to both Cdc11 and Cdc3 [9].

In the filamentous fungus *Aspergillus nidulans* four of the five septins (AspA, AspB, AspC and AspD) are orthologs of the *S. cerevisiae* core septins and localize as a ring or collar to the region where the hypha emerges from the conidium [10], [11], [12]. AspA is orthologous to *S. cerevisiae* Cdc11. AspB is orthologous to Cdc3. AspC is orthologous to Cdc12 and AspD is orthologous to Cdc10. Loss of AspC prevents the proper localization of AspA and visa versa consistent with participation in a heteropolymeric septin complex like that in *S. cerevisiae* [13].

## Materials and Methods

### Strains and growth conditions

Yeast strains and plasmids used in this study are listed in Tables 1, 2 and 3. Yeast strains were incubated in YPD (rich medium), SC (synthetic complete medium with amino acids omitted as necessary for plasmid selection), sporulation medium or SLAD (sigma strains) [14] at 30°C unless otherwise stated. Plasmids were constructed using standard methods [14], [15]. *A. nidulans* AspC was amplified from cDNA using the Advantage High Fidelity 2 PCR kit (Clontech Laboratories, Inc, Mountain View, CA), cloned into PCR 2.1 TOPO (Invitrogen Corp., Carlsbad, CA) and sequenced. For expression in *S. cerevisiae*, *aspC* was cloned behind the inducible *GAL1* promoter or the constitutive *ADH1* promoter [16], [17] to yield plasmids pRL16 (*GAL1*-AspC) and pRL19 (*ADH1*-AspC). Plasmids were introduced into *S. cerevisiae* by transformation using LiAc [18].

**Table 1:** *S. cerevisiae* strains used in this study.

Strain	Relevant genotype	Source
ARL7	Sc295 with pRL16 (*P_GAL1_*-*aspC*, *URA3*)	this study
ARL15	Sc295 with pRS316GU (empty vector)	this study
ARL21	BY4741 with pRS316GU (empty vector)	this study
ARL22	BY4741 with pRL16 (*P_GAL1_*-*aspC*, *URA3*)	this study
ARL67	BY4741with pRL20 (*P_GAL1_*-*aspC)* and pJT2044 (*CDC12-GFP*)	this study
ARL68	BY4741 with pRL19 (*P_ADH1_-aspC1*, *URA3*)	this study
AYH3	Sc295 with pRL16 (*P_GAL1_*-*aspC*, *URA3*)	this study
AYH17	BY4741 with pHY33 (*P_GAL1_*-*GFP*-*aspC)*	this study
AYH37	*CDC12/cdc12Δ* with pYH33 (*P_GAL1_*-*GFP*-*aspC)*	this study
AYH38	*CDC12/cdc12Δ* with pYH35 (*P_ADH1_*-*GFP*-*aspC)*	this study
AYH39	*CDC12/cdc12Δ* with pYH42 (*P_ADH1_-aspC)* and pJT2044 (*CDC12-GFP*)	this study
BY4741	***a*** * his3Δ1 leu2Δ0 met15Δ0 ura3Δ0*	Open Biosystems
BY4742	***α*** * his3Δ1 leu2Δ0 lys2Δ0 ura3Δ0*	Open Biosystems
BY4743	***a/α*** * 4741/4742*	Open Biosystems
*CDC12/cdc12Δ*	BY4743 background	Open Biosystems
DDY1453	**a** *ura3-52 his7 ade2 trp1-289 cdc3-1ts*	J. Thorner [3], [30]
DDY1462	**a** *ura3-52 cdc12-6ts*	J. Thorner
DDY1455	**a** *ura3-52 his7 tyr1 ade2 lys2 cdc11-1ts*	J. Thorner [3], [30]
DDY1476	**a** *ura3-52 trp1-289 ade2 lys2 tyr1 cdc10-1ts*	J. Thorner
MLY40	**α** *ura3-52* (congenic with *Σ1278b*)	J. Heitman [26]
MLY61	**a/α** *ura3-52/ura3-52* (congenic with *Σ1278b*)	J. Heitman [26]
Sc295*	**a** *ura3-52 leu2-3 112 reg1-501 gal1 pep4-3*	W. Schmidt [15], [31], [32]
SY3721	**α** *ura3 axl1::HIS3*	W. Schmidt [33]
Y187	*α ura3-52 his3-200 ade2-101 trp1-901*	Clontech

**Table 2:** *S. cerevisiae* plasmids used in this study.

Plasmid	Description	Source
pCR2.1-TOPO	PCR cloning vector	Invitrogen
pGBKT7	*P_ADH1_*, DNA-BD, *TRP1*	Clontech
pJT2044/MVB89	*CEN, URA3, CDC12-GFP*	J. Thorner
pQBI25	sgGFP	Quantum Inc.
pRL16	*P_GAL1_*-*aspC*, *URA3*	this study
pRL19	*P_ADH1_-aspC1*, *URA3*	this study
pRL20	*P_GAL1_*-*aspC*, *LEU2*	this study
pRS316	*CEN, URA3*	W. Schmidt [15]
pRS316GU	*CEN*, *URA3, P_GAL1_*	W. Schmidt [15], [31], [32]
pYH33	*CEN*, *URA3*, *P_GAL1_*-*GFP*-*aspC*	this study
pYH35	*CEN*, *URA3, P_ADH1_-GFP-aspC*	this study
pYH42	*P_ADH1_-aspC*, *LEU2*	this study

**Table 3:** AspC-induced phenotype in *S. cerevisiae* deletion mutants.

*Deletion^a^*	*Phenotype^b^*	*Function^c^*
*Afr1Δ* d e	PS	Cdc12i, Shmoo
*bem4Δ* d	PS	Cdc12i
*Bni1Δ* d e	N	Cdc12i, Formin
*Bnr1Δ* d e	PS	Formin
*CDC3/cdc3Δ* d	PS	Septin
*cdc10Δ* d	Y	Septin
*CDC11/cdc11Δ* d	PS	Septin
*CDC12/cdc12Δ* d e	PS	Septin
*Cla4Δ* d	PS	Cdc12i, Cell Pol
*Dfg16Δ* d	PS	Hap Inv
*Flo8Δ* d	PS	cAMP
*Flo11Δ* d	PS	cAMP
*Gic1Δ* d e	PS	Cdc12i, Cell Pol
*Gic2Δ* d e	PS	Cdc12i, Cell Pol
*Kss1Δ* d	PS	MAPK
*Ras2Δ* d	PS	Pseudohyphal
*Shs1Δ* d	PS	Septin
*Spr3Δ* d	PS	Septin
*Spr28Δ* d	PS	Septin
*Ste7Δ* d	PS	MAPK
*Ste11Δ* d	PS	MAPK
*Ste20Δ* d	PS	MAPK
*Swe1Δ* d e	PS	Morph Ckpt
*Tec1Δ* d	PS	MAPK

a deletion mutants from Open Biosource.

b *S. cerevisiae* deletion mutants were transformed with *A. nidulans aspC* behind the *GAL1* inducible or the *ADH1* constitutive promoter as indicated. Phenotype after galactose induction (*GAL1* promoter) or with no induction (*ADH1* promoter) is indicated. PS = pseudohypha-like cells in >25%; Y = yeast; N = novel phenotype.

c cAMP, cAMP pathway component; Cdc12i, Cdc12-interacting (genetic or biochemical); Cell Pol, establishment of cell polarity; Hap Inv, Haploid invasive growth; MAPK, MAPK pathway component; Morph Ckpt, morphogenesis checkpoint component; Pseudohyphal, diploid pseudohyphal growth; shmoo, mating projection formation.

d *GAL1* promoter.

e *ADH1* promoter.

### Induction of filamentous growth and microscopy

Haploid Sc295 was transformed with pRL16 (*GAL1*-AspC) and pRS316 (empty vector). Single transformant colonies were transferred to liquid selective medium, grown to an OD_600_ of 0.4 and split into two tubes. One tube was induced by the addition of fresh medium with galactose (0.5% final concentration) while the second tube received fresh medium without galactose. Strains 4741 and 4743 were transformed with pRL19 (*ADH1*-AspC) and plated on selective medium without galactose. Cells were incubated for 24 or 48 hours and fixed using standard techniques [19]. MLY40 and MLY61, derivatives of Σ1278b, were transformed with pJT2044 (cdc12-GFP), pYH35 (*ADH1*-AspC-GFP), and pRS316 (empty vector) and grown in SLAD liquid and solid medium. Samples were incubated overnight at 30°C in appropriate medium. Overnight cultures were used to inoculate fresh medium, incubated at 30°C for 2hrs and observed using a Zeiss Axioplan microscope. Digital images were acquired using an Optronics digital imaging system and were prepared using Adobe Photoshop cs version 8.0.

### Quantitation of morphological phenotypes

All morphology counts were performed at least twice by two or more investigators counting a minimum of 300 cells each. Counts were very similar in all cases. The average count is presented.

### Invasive growth assay

ARL15 (pRS316GU in Sc295), AYH3 (pRL16 in Sc295), ARL21 (pRS316 in 4741), ARL22 (pRL16 in 4741), Sc295 and SY3721 (haploid invasive strain) were inoculated to SC –ura solid medium with and without 0.5% galactose. The above strains plus haploid strain 4741 were inoculated to YPD with and without 2% galactose. All strains were incubated for three days at 30°C. On the third day plates were washed with gently running water and rubbed with a gloved finger [20]. Photos were taken with a Kodak CX6330 digital camera. Experiments were repeated three times with identical results.

### Pathway mutant studies

We obtained seventeen haploid deletion strains (*afr1Δ*, *bem4Δ*, *bni1Δ*, *bnr1Δ*, *bud8Δ*, *cla4Δ*, *dfg16Δ*, *flo8Δ*, *flo11Δ*, *gic1Δ*, *gic2Δ*, *kss1Δ*, *ras2Δ*, *ste7Δ*, *ste11Δ*, *ste20Δ*, *swi1Δ*, *tec1Δ*) [21] through Open Biosystems (AL). We confirmed the identity of all deletion strains by PCR amplification of unique tags using sequences provided by the Saccharomyces Genome Deletion Project web page (www-sequence.stanford.edu/group/yeast_deletion_project/deletions3.html). Five colonies from a streaked plate of each deletion strain were tested using colony PCR with modifications to protocols from the Stanford Yeast Deletion website. Modifications included making a master mix of diluted primer A and KanB for 5 individual Easy Start Micro 20 PCR tubes (Molecular Bioproducts, San Diego, CA). The final primer concentration in each tube was 0.37 µM. A pipette tip was used transfer 0.25 µL of cells (or less) from a yeast colony to the PCR tube. PCR was carried out with a RoboCycler Gradient 96 thermocycler (Stratagene, La Jolla, CA) under the following conditions: 1 cycle at 94C for 4 min, 35 cycles at 94C for 30 seconds, 64C for 1 min, and 72C for 2 min, and 1cycle at 72C for 10 min. The total contents of each tube were electrophoresed on a 1% TBE gel with a 100 bp DNA ladder (Promega, Madison, WI). PCR products amplified from all five colonies were the expected size in all strains. These haploid deletion strains were transformed with pRS316 (empty vector control) and pRL16 (pRS316 with AspC) and plated on selective medium. For induction, single colonies were grown in selective liquid medium to an optical density at A_600_ of 0.4; the sample was split and centrifuged. Half of the sample was resuspended in two volumes of fresh medium; the other half was resuspended in two volumes of fresh medium with 0.5% galactose and no glucose. Samples were incubated overnight (16 hrs) and examined microscopically. Most deletion strains were also transformed with pRL19 (*ADH1*-AspC) and plated on selective medium. We obtained this vector by cloning the constitutively active *ADH1* promoter into *Sal*I and *Kpn*I restriction sites in pRL16 (*GAL1*-AspC). Transformation and induction experiments for each strain were repeated at least twice.

### GFP Localization

A triple GFP mutant pQBI25 (Quantum, Inc.) was fused inframe to the N-terminus of AspC in pRS316 using the *Sal*I-*Eco*RI restriction sites to give pYH33. The constitutively active *ADH1* promoter from pRL19 was cloned into the *Kpn*I and *Sal*I restriction sites to drive fusion protein expression to yield plasmid pYH35 (*ADH1*-GFP-AspC). For co-transformations the *LEU2* marker was cloned into an *Nco*I site in the middle of the *URA3* marker of pRL19 (*ADH1*-AspC, *URA3*) to get pRL20 (*ADH1*-AspC, *LEU2*). We transformed both pJT2044 (cdc12-GFP, *URA3*) and pRL20 (*ADH1*-AspC, *LEU2*) into the diploid yeast strain *CDC12/cdc12Δ.* In separate experiments *CDC12/cdc12Δ* was transformed with pYH35 (*ADH1*-GFP-AspC). All transformants were incubated overnight at 30°C. Overnight cultures were transferred to fresh medium, incubated at 30°C for 2 hrs and observed using a Zeiss Axioplan microscope. Digital images were acquired using an Optronics digital imaging system and were prepared using Adobe Photoshop cs version 8.0.

### Septin deletion mutants

We obtained haploid (*cdc10Δ,sep7Δ, spr3Δ, spr28Δ*) and heterozygous diploid (*CDC3/cdc3Δ, CDC11/cdc11Δ* and *CDC12/cdc12Δ*) *S. cerevisiae* septin deletion strains [21] through Open Biosystems (AL). Haploid deletion strains were transformed with pRS316 (control), pRL16 (*GAL1*-AspC) and pRL19 (*ADH1*-AspC) vectors and plated on selective medium. Strains with *GAL1*-AspC were induced as noted above in pathway mutant studies with fresh medium with or without 0.5% galactose. The diploid deletion strain *CDC12/cdc12Δ* was transformed with pYH35 (*ADH1*-GFP-AspC) or with both pJT2044 (Cdc12-GFP) and pRL20 (*ADH1*-AspC) and grown on selective media. Transformation and induction experiments for each strain were repeated at least twice. Counts of filaments vs. buds were made for each strain.

### Sporulation

Diploid heterozygous septin deletion strains, with and without AspC were inoculated to sporulation medium [14] and incubated at room temperature for 4–7 days. Colonies were transferred to a slide with mounting solution and examined microscopically for ascospores. Septin deletion strain *CDC12/cdc12Δ* was transformed with *ADH1*-GFP-AspC and incubated on selective medium. Single colonies were streaked and transferred to sporulation medium [14] and incubated at room temperature for 4–7 days. For tetrad analysis, a micromanipulator was used to transfer ascospores to YPD plus sorbitol medium, then to selective medium and colonies were scored.

## Results and Discussion

As part of a protein interaction screen, we cloned septins from the filamentous fungus *A. nidulans* into a two-hybrid bait vector and expressed them in the budding yeast *S. cerevisiae*. Interestingly, expression of *aspC*, the ortholog of *CDC12* that occupies the central position in the yeast core septin complex, changed the appearance of *S. cerevisiae* on solid medium from the round, smooth colonies typical of yeast growth to the larger, fuzzy colonies typical of hyphal growth. Microscopic examination of *S. cerevisiae* transformants bearing *aspC* showed many highly elongated, filament-like cells. No other *A. nidulans* septin caused such a dramatic shift in the morphology of *S. cerevisiae*. When an extra copy of *CDC12* driven by its own promoter was introduced into *S. cerevisiae* it failed to alter the budding phenotype of either haploid or diploid strains (data not shown) consistent with previous work showing that *CDC12* driven by the *GAL1* promoter does not induce filamentation, nor does it perturb the bud-neck localization of septins Cdc3, Cdc10 and Cdc11 [22].

**Figure 1:**
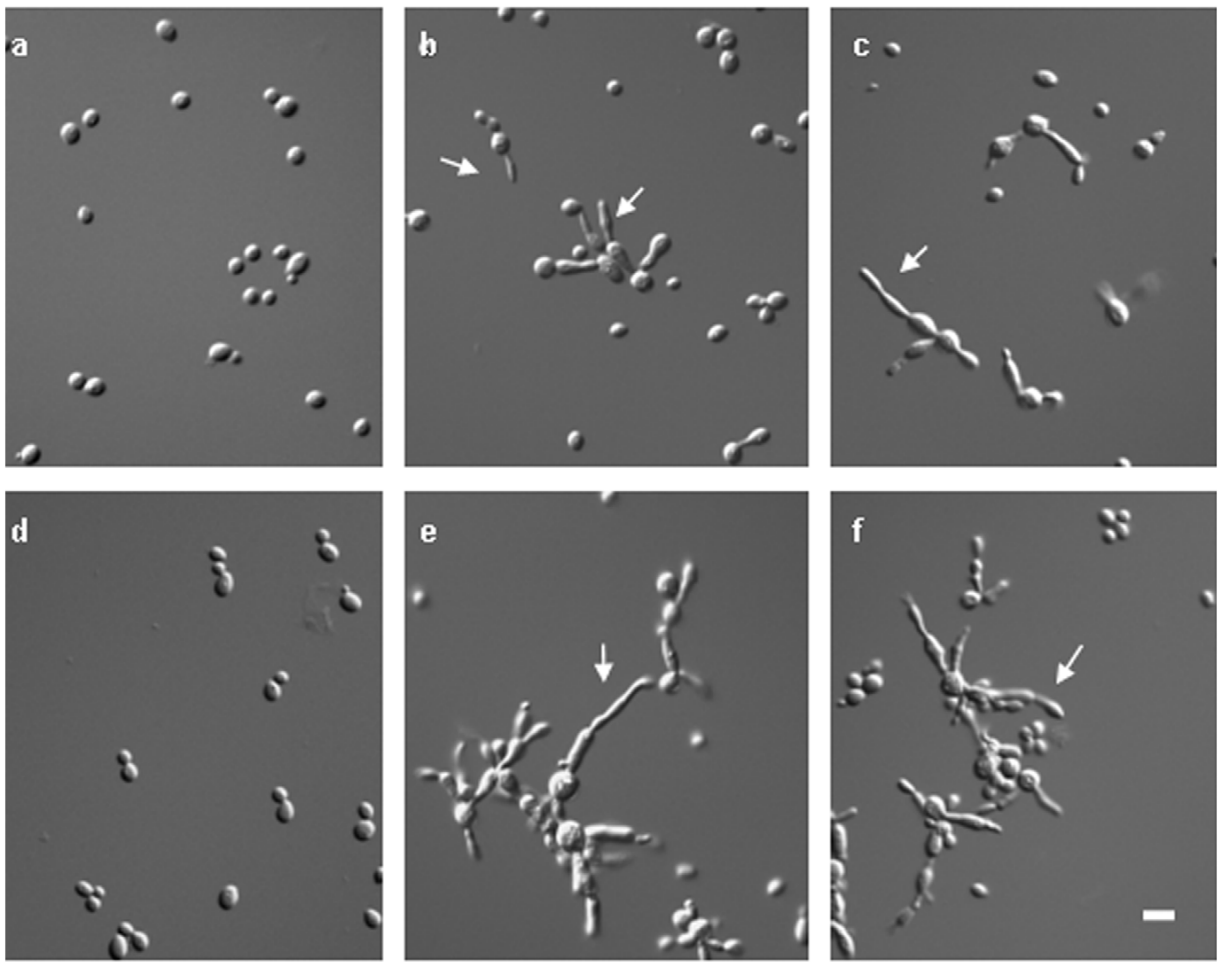
*A. nidulans* septin AspC induces filaments in *S. cerevisiae*. *S. cerevisiae* transformed with the *A. nidulans* septin gene *aspC* under the control of the inducible *GAL1* promoter and incubated for 24 (a–c) or 48 hr (d–f). a, d) uninduced cultures grow only as budding yeasts. b, c, e, f) induced cultures grow as yeasts and atypical pseudohyphae. Arrows indicate examples of atypical pseudohyphae. Scale bar = 10 µ.

**Figure 2:**
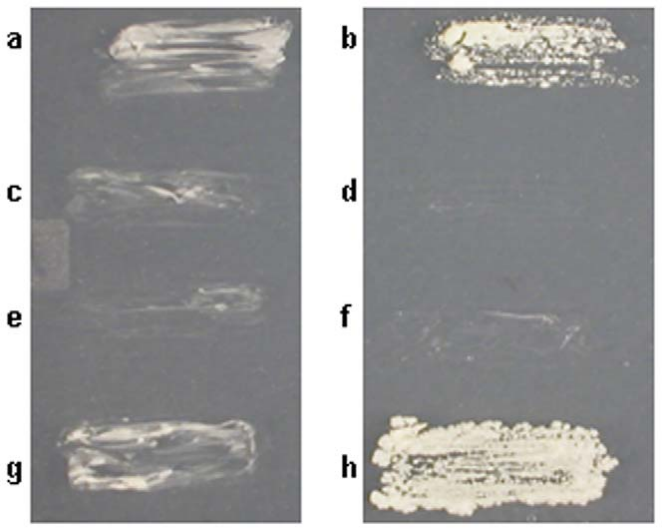
*A. nidulans aspC* partially complements *S. cerevisiae cdc3* and *cdc12* mutants. *S. cerevisiae* temperature-sensitive septin mutants were transformed with an empty vector (pRS316) or the *A. nidulans aspC* gene under the control of the constitutive *ADH-1* promoter (pRL19) and incubated for 24 hr at 37°C. a, b) *S. cerevisiae cdc3*. c, d) *S. cerevisiae cdc10*. e, f) *S. cerevisiae cdc11*. g, h) *S. cerevisiae cdc12*. a, c, e, g) transformed with empty vector. b, d, f, h) transformed with the *A. nidulans aspC* gene under the control of the constitutive *ADH-1* promoter.

To further investigate the morphological changes *aspC* induced, we created multiple *S. cerevisiae* plasmids and strains using standard methods (Table 1, 2) [14], [15], [23]. When the *A. nidulans* septin *aspC* was expressed in *S. cerevisiae* strain Sc295 driven by the *GAL1* promoter (ARL7 and AYH3) 54% of the cells formed highly elongated filament-like extensions rather than buds (Fig. 1). AspC also induced highly elongated, filament-like cells in strain BY4741 driven by either *GAL1* (ARL22) or the constitutive *ADH1* promoter (ARL68) (data not shown). To determine whether AspC could substitute for the *S. cerevisiae* septins, we transformed temperature-sensitive *cdc3*, *cdc10*, *cdc11* and *cdc12* yeast strains with *aspC* driven by the constitutive *ADH1* promoter. *A. nidulans aspC* complemented the temperature sensitivity of *cdc3* and *cdc12* mutants (Fig. 2), though microscopic examination of transformants showed cells were still abnormally elongated. This partial complementation showed that the *A. nidulans* septin can at least partially substitute for two of the *S. cerevisiae* core septins.

**Figure 3:**
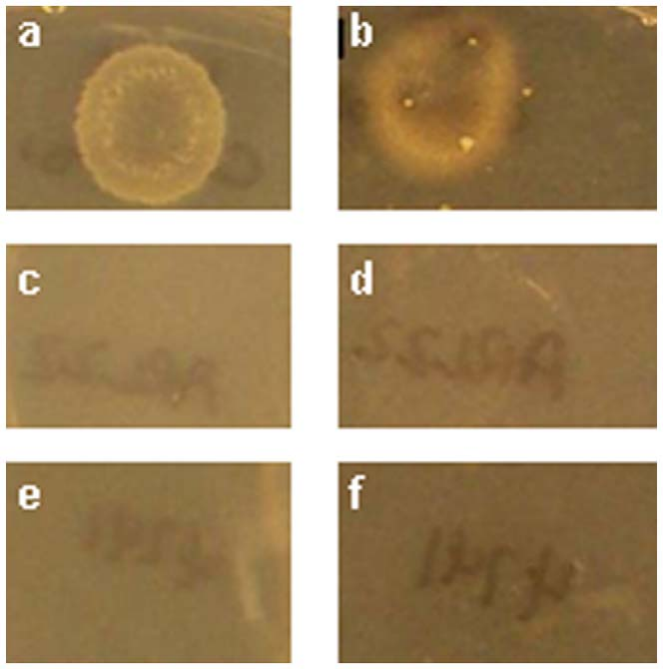
AspC-induced atypical pseudohyphae are not invasive. *S. cerevisiae* strains known to be haploid invasive (SY3721), noninvasive (BY4741) and transformed with the *A. nidulans* septin gene *aspC* under the control of the inducible *GAL1* promoter (ARL22) were inoculated to solid medium with and without 0.5% galactose, incubated 3d at 30°C and washed with gently running water and rubbed with a gloved finger. a, b) SY3721. c, d) ARL22. e, f) BY4741. a, c, e) Noninducing medium. B, d, f) Inducing medium.

Laboratory strains of *S. cerevisiae* generally grow as budding yeasts; however certain strains, such as Σ1278b, form pseudohyphae (highly elongated, filament-like cells that invade agar) when incubated on low nitrogen solid medium [24]. Similarly, haploids of certain strains also form invasive pseudohyphae when incubated on solid rich medium [20]. Both diploid pseudohyphal and haploid invasive growth require Flo11, a GPI-anchored cell surface glycoprotein that is transcriptionally regulated by the cAMP pathway (via Flo8) and the MAPK pathway (via Ste12 and Tec1)[25]. To determine whether the highly elongated, filament-like cells that we observed when *aspC* was expressed in *S. cerevisiae* were invasive, we compared them to strains that are known to form pseudohyphae (MLY40 and MLY61 derivatives of Σ1278b) or to be haploid invasive (SY3721) in an invasive growth assay on solid medium [20], [26]. The elongated, filament-like cells induced by the *A. nidulans* septin AspC in *S. cerevisiae* did not invade agar (Fig. 3). To determine whether AspC induced the highly elongated filament-like cells via the previously described cAMP or MAPK Flo11-dependent pseudohyphal or haploid invasive pathways [27], we introduced *A. nidulans aspC* into nine *S. cerevisiae* haploid strains with deletions in genes important for these pathways. AspC induced pseudohypha-like cells in all cAMP, MAPK and invasive growth deletion strains tested, including *flo11Δ* (Table 3). Additionally, the *S. cerevisiae* wildtype strains used in this study are derived from S288C, which has previously been shown to be unable to undergo haploid invasive or pseudohyphal growth because of a mutation in *FLO8* [20]. AspC induction of pseudohypha-like cells in strains deleted for genes known to be required for pseudohyphal and haploid invasive growth and in S288C derivates is consistent with formation via an alternate pathway. Because the AspC-induced elongated, filament-like cells in *S. cerevisiae* differed from previously described pseudohyphae in several ways (they formed in liquid and solid media, on rich and minimal media, did not invade agar and did not require previously identified pseudohyphal pathway genes), we refer to them as “atypical pseudohyphae.”

The *S. cerevisiae* septins Cdc3, Cdc10, Cdc11 and Cdc12 form the core heteropolymeric septin complex at the base of the bud. Disruption of any one of these septins leads to mislocalization of the others. To determine whether induction of atypical pseudohyphae by the *A. nidulans* septin AspC requires the *S. cerevisiae* septins, we introduced *aspC* into *S. cerevisiae* haploid strains missing nonessential septins (*cdc10Δ, sep7Δ, spr3Δ, spr28Δ*) obtained through Open Biosystems (AL) [21]. Haploid deletion strains were transformed with either pRS316 (control), pRL16 (*P_GAL1_*-*aspC*) or pRL19 (*P_ADH1_*-*aspC*). Expression of *aspC* induced atypical pseudohyphae in yeast deleted for those septins that are not part of the core heteropolymeric septin complex (*shs1Δ, spr3Δ* and *spr28Δ*) (Table 3). However, *aspC* did not induce atypical pseudohyphae in *cdc10Δ*. The failure of *aspC* to alter morphology in *cdc10Δ* suggested that the *A. nidulans* septin AspC might interact with the *S. cerevisiae* core heteropolymeric septin complex at the neck to induce atypical pseudohyphae.

**Figure 4:**
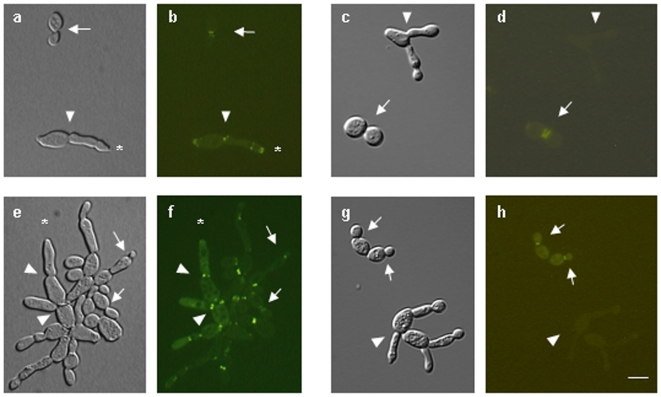
*A. nidulans* AspC-GFP localizes to the bases of buds and to the bases and tips of atypical pseudohyphae while *S. cerevisiae* Cdc12-GFP localizes exclusively to the bases of buds. a,b,e,f) *S. cerevisiae* was transformed with the *A. nidulans* septin *aspC* fused to GFP under the control of the constitutive *ADH1* promoter. c,d,g,h) *S. cerevisiae* was transformed with the *A. nidulans* septin *aspC* under the control of the constitutive *ADH1* promoter and the *S. cerevisiae* septin *CDC12* fused to GFP. The *A. nidulans* septin is visible in both yeasts and atypical pseudohyphae while the *S. cerevisiae* septin is visible only in yeasts. Arrows indicate examples of bud necks. Arrowheads indicate examples of atypical pseudohyphae necks. Asterisks indicate examples of atypical pseudohyphae tips. Scale bar = 10 µ.

To determine if the *A. nidulans* septin AspC localized to the base of buds or atypical pseudohyphae, we constructed plasmids bearing GFP fused to AspC and driven by the *GAL1* (pYH33) or *ADH1* promoter (pYH35) and introduced them into haploid (AYH17) or heterozygous *CDC12/cdc12Δ* diploid (AYH37, AYH38) strains. AspC-GFP induced atypical pseudohyphae in both haploid and diploid strains just as AspC without the GFP tag had. AspC-GFP label was seen with similar frequency at the bases of yeasts and of atypical pseudohyphae (Fig. 4, a, b, e, f). Label was also sometimes seen at the tips of atypical pseudohyphae (Fig. 4b, f).

To determine whether the *A. nidulans* septin AspC affected the localization of the orthologous yeast septin Cdc12, we introduced *aspC* with no label and *CDC12-GFP* into haploid (ARL67) and *CDC12/cdc12Δ* heterozygous diploid strains (AHY39). Surprisingly, in both the haploid and diploid strains Cdc12-GFP localized to the base of buds, but not of atypical pseudohyphae (Fig. 4d, h). Quantitation of Cdc12-GFP localization verified that virtually all labeled Cdc12 was at the necks of yeast (99.5%, n = 300) and virtually none was at the necks of atypical pseudohyphae (0.5%, n = 300).

**Figure 5:**
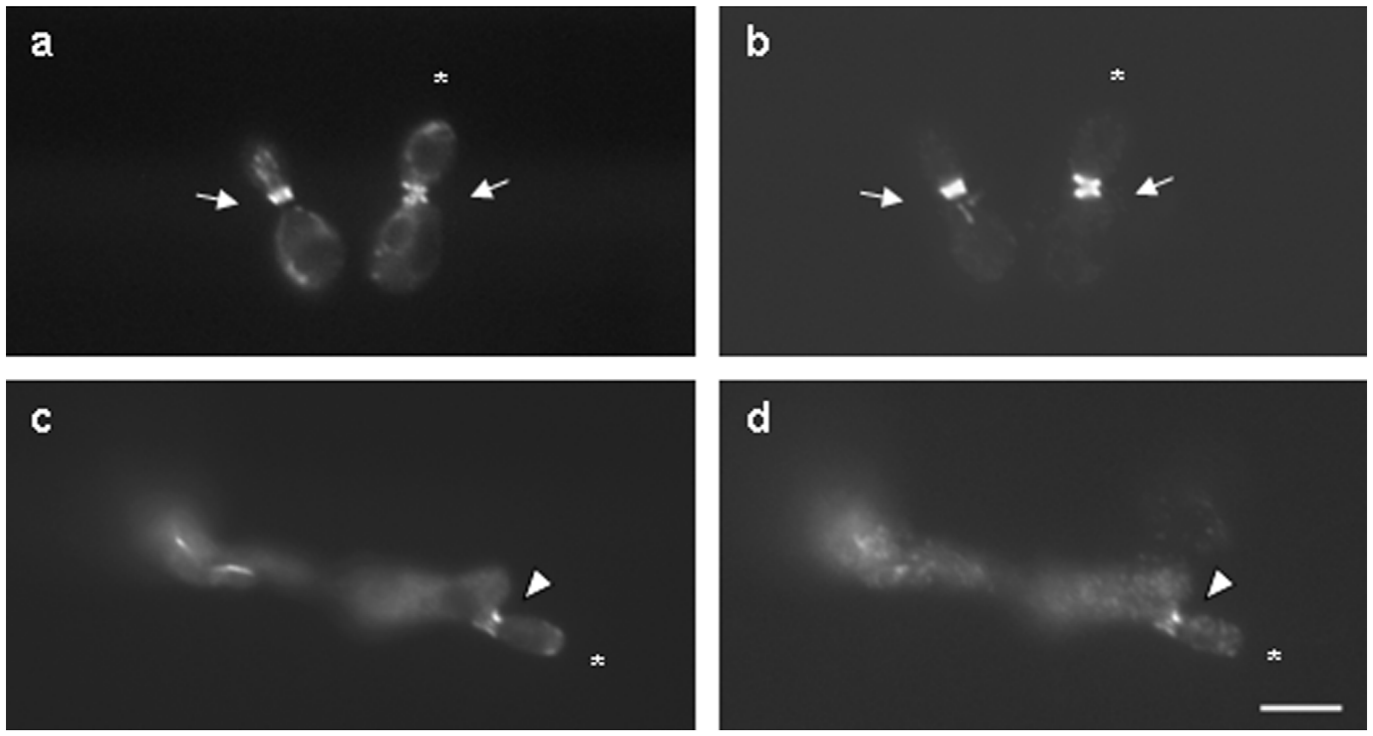
*A. nidulans* AspC-GFP co-localizes with *S. cerevisiae* Cdc11 to bud and atypical pseudohyphae necks. *S. cerevisiae* was transformed with the *A. nidulans* septin *aspC* fused to GFP under the control of the constitutive *ADH1* promoter and incubated for 48 hrs. a, c) AspC-GFP. b, d) Cdc11 detected by a commercially available primary antibody and rhodamine-coupled secondary antibody. Arrows indicate examples of bud necks. Arrowheads indicate examples of atypical pseudohyphae necks. Asterisks indicate examples of bud and atypical pseudohyphae tips. Scale bar = 10 µ.

To determine whether the *A. nidulans* septin AspC affected the localization of members of the native yeast septin scaffold other than Cdc12, we used commercially available antibodies to label Cdc11 in a *S. cerevisiae CDC12/cdc12Δ* heterozygous diploid strain carrying *P_ADH1_- aspC-GFP* (AYH38). We found co-localization of AspC-GFP and Cdc11 at bases of both yeasts and atypical pseudohyphae (Fig. 5) suggesting that the septin scaffold is intact when AspC is present in both yeasts and atypical pseudohyphae, despite the absence of Cdc12 in atypical pseudohyphae. We also observed AspC-GFP, but not Cdc11, at the tips of many buds and atypical pseudohyphae. The presence of Cdc12-GFP exclusively at bud necks and of AspC-GFP and Cdc11 at both bud and atypical pseudohypha necks suggests that AspC might compete with Cdc12 for incorporation into the septin scaffold at the neck and that the substitution of AspC for Cdc12 induces atypical pseudohyphae.

**Figure 6:**
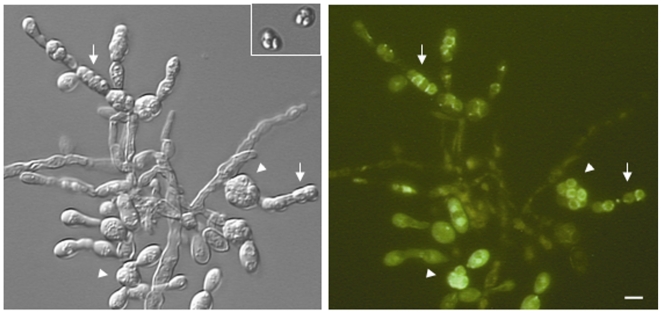
*A. nidulans* AspC induces abnormal asci in *S. cerevisiae*. *S. cerevisiae* was transformed with the *A. nidulans* septin *aspC* fused to GFP under the control of the constitutive *ADH1* promoter and induced to sporulate. Arrows indicate examples of linear asci. Arrowheads indicate examples of round asci. Inset shows normal yeast tetrad ascus for comparison. Scale bar = 10 µ.

The *A. nidulans aspC* septin also induced abnormal morphology during sexual reproduction in *S. cerevisiae*. When diploid *S. cerevisiae* is placed on sporulation medium it undergoes meiosis and packages four spores into an ascus (a round protective sac). When *S. cerevisiae CDC12/cdc12Δ* carrying GFP-AspC was induced to sporulate, asci were enlarged with spores arranged in long chains or clusters reminiscent of the asci of filamentous fungi (Fig. 6). Over half (65%) of the resulting asci contained more than four spores, with as many as twelve seen in some cases. Five apparently normal tetrad asci and five abnormal enlarged asci were dissected. Each of the normal tetrads had two viable spores while no spores from abnormal asci were viable (data not shown). Thus *aspC* cannot substitute for *CDC12* during sexual reproduction, in contrast to its ability to partially substitute for cdc12 during vegetative growth of the *cdc12* ts mutant.

**Figure 7:**
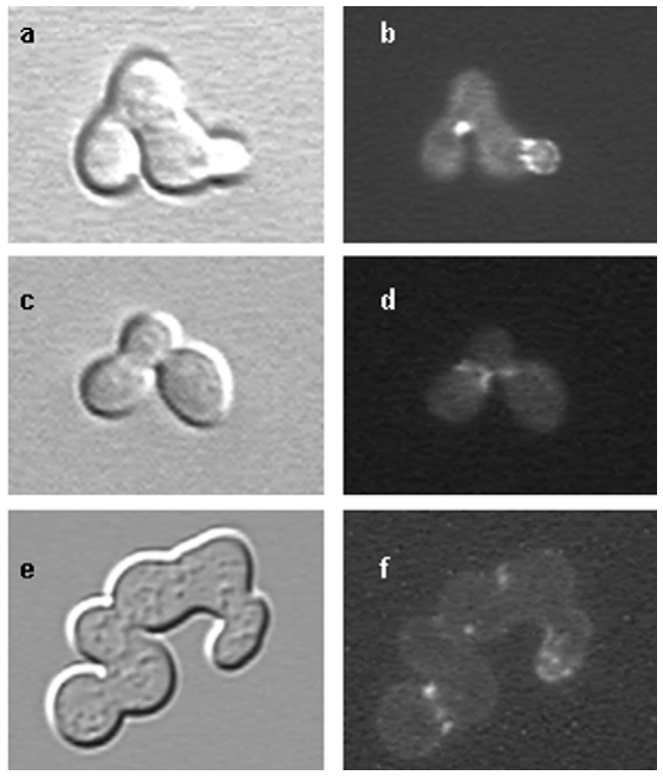
*S. cerevisiae bni1Δ* transformed with *A. nidulans aspC* forms triads or short chains of yeast cells. *S. cerevisiae bni1* ***Δ*** was transformed with the *A. nidulans* septin *aspC* fused to GFP under the control of the constitutive *ADH1* promoter and incubated for 48 hrs. a,c,e) phase contrast and DIC images. b, d, f) aspC-GFP.

Given the scaffolding role of septins, a likely mechanism for AspC-induced morphological changes is inappropriate recruitment or regulation of proteins that localize in a septin-dependent manner. To investigate this possibility, we introduced *aspC* into seven *S. cerevisiae* strains with deletions in genes encoding Cdc12-interacting proteins or a morphogenesis checkpoint component (*AFR1*, *BEM4*, *BNI1*, *CLA4*, *GIC1*, *GIC2* and *SWE1*) [5]. Six of seven deletion strains formed atypical pseudohyphae upon introduction of GFP-AspC (Table 3). The one exception, *bni1Δ*, made triads or short chains of yeast cells with broader necks and GFP-AspC bars or dots in neck regions and at bud tips (Fig. 7). Introduction of extra *CDC12* into *bni1Δ* carrying GFP-AspC restored a normal budding phenotype to virtually all cells, consistent with the notion that AspC competes with Cdc12 for incorporation into the septin scaffold and that AspC inappropriately recruits Bni1. In control experiments, *bni1Δ* transformed with Cdc12-GFP made normal budding cells with necks slightly broader than those of wildtype and Cdc12-GFP localizing to neck rings (data not shown).

The formins Bnr1 and Bni1 nucleate actin filament assembly in *S. cerevisiae* [28]. Though the functions of yeast formins overlap, Bnr1 assembles actin cables that form a stable axis between mother and daughter, while Bni1 assembles dynamic actin cables that target vesicles to multiple locations in the bud and are required for polar growth [28]. To determine whether both formins might be required for AspC-induced filamentation, we introduced *aspC* into *bnr1Δ* (Table 3). AspC induced atypical pseudohyphae in *bnr1Δ* showing that a stable mother/bud axis is not required for filament formation. The failure of AspC to induce atypical pseudohyphae in *bni1Δ* suggests that the dynamic actin cables used in polar growth of buds are also used in polar growth of atypical pseudohyphae.

The morphology of many fungal species is very plastic, changing dramatically in response to changes in the cellular environment. This plasticity is thought to enhance the survival of fungi by allowing them to alter their growth modes to better cope with hostile environments. There is increasing evidence from a variety of systems that septins play critical roles in directing cell shape [29]. Because they form scaffolds that recruit and organize many other proteins, including those that regulate morphogenesis and cell cycle [5], it seems reasonable that changes in septins would have large effects on cell shape by virtue of their binding partners. Our results suggest that septins play an important role in the morphological plasticity of fungi.
